# Efficient Synthetic Defect on 3D Object Reconstruction and Generation Pipeline for Digital Twins Smart Factory

**DOI:** 10.3390/s25226908

**Published:** 2025-11-12

**Authors:** Viet-Hoan Nguyen, Thi-Ngot Pham, Jun-Ho Huh, Pil-Joo Choi, Young-Bong Kim, Oh-Heum Kwon, Ki-Ryong Kwon

**Affiliations:** 1 R&D Center, Intown Co., Ltd., Busan 08592, Republic of Korea; viethoan_nvh@pukyong.ac.kr; 2 Department of Artificial Intelligence Convergence, Pukyong National University, Busan 48513, Republic of Korea; 3 Department of Data Informatics, Korea Maritime & Ocean University, 727, Taejong-ro, Yeongdo-gu, Busan 49112, Republic of Korea; 4 Interdisciplinary Major of Ocean Renewable Energy Engineering, Korea Maritime & Ocean University, 727, Taejong-ro, Yeongdo-gu, Busan 49112, Republic of Korea

**Keywords:** synthetic data generation, NERF, 3D model reconstruction, object detection, YOLO

## Abstract

High-quality 3D objects play a crucial role in digital twins, while synthetic data generated from these objects have become essential in deep learning-based computer vision applications. The task of collecting and labeling real defects on industrial object surfaces has many challenges and efforts, while synthetic data generation feasibly replicates huge amounts of labeled data. However, synthetic datasets lack realism in their rendered images. To overcome this issue, this paper introduces a single framework for 3D industrial object reconstruction and synthetic defect generation for digital twin smart factory applications. In detail, NeRF is applied to reconstruct our custom industrial 3D objects through videos collected by a smartphone camera. Several NeRF-based models (i.e., Instant-NGP, Nerfacto, Volinga, and Tensorf) are compared to choose the best outcome for the next step of defect generation on the 3D object surface. To be fairly evaluated, we train four models using the Nerfstudio framework with our three custom datasets of two objects. From the experiment’s results, Instant-NGP and Nerfacto achieve the best outcomes, outperforming all other methods significantly. The exported meshes of 3D objects are refined using Blender before loading into NVIDIA Omniverse Code to generate defects on the surface with the Replicator. To evaluate the object detection performance and to verify the benefits of synthetic defect data, we conducted experiments with YOLO-based models on our synthetic and real-plus-synthetic defects. From the experiment’s results, the synthetic defect data contribute to improving YOLO models’ generalization capability with the highest and lowest accuracy mAP@0.5 enhancement of 18.8 and 1.5 percent on YOLOv6n and YOLOv8s, respectively.

## 1. Introduction

The growing complexity of industrial manufacturing has highlighted the need for robust quality control, particularly defect inspection, as product defects can lead to substantial economic losses, reduced customer satisfaction, and safety risks [1]. However, the development of effective automated defect inspection systems is limited by the scarcity of annotated defect image datasets, which are essential for training Convolutional Neural Network (CNN) models. Manual annotation remains time-consuming and expensive, posing a major barrier to the widespread deployment of AI-based inspection technologies [2].

To overcome this challenge, researchers have increasingly adopted synthetic data generation using 3D software and simulation environments such as Unity [3], Unreal Engine [4], NVIDIA Omniverse [5], and Blender [6]. These platforms enable the creation of large, diverse, and precisely labeled datasets at low cost, allowing the simulation of rare or hazardous defect scenarios that are difficult to capture in real settings. Such synthetic datasets significantly improve the scalability and robustness of deep learning models for defect detection.

A key challenge, however, lies in obtaining accurate 3D models of industrial objects. While Computer-Aided Design (CAD) data are commonly available, licensing restrictions often limit their use for rendering or dataset generation. As an alternative, image-based 3D reconstruction techniques—using images captured from multiple viewpoints—offer a practical and flexible solution for creating realistic 3D models without relying on restricted CAD data. This work aims to address two challenges by proposing a unified framework that integrates 2D image-based 3D object reconstruction with synthetic defect generation for developing defect inspection systems. By combining these two techniques, the proposed framework expects to overcome the limitations imposed by CAD licensing restrictions and the scarcity of annotated defect images. The incorporation of synthetic data into training deep learning-based object detection models for industrial defect inspection application is expected to significantly improve the accuracy and reliability of defect detection, ultimately enhancing the effectiveness and efficiency of our system in diverse industrial smart factory applications.

In the realms of computer vision and photogrammetry, precise 3D reconstruction plays a crucial role in applications such as defect inspection, reverse engineering, structural health monitoring (SHMS), and digital heritage preservation. The growing demand for cost-effective and portable solutions with high geometric precision has driven the development of two main approaches: LiDAR-based and image-based methods [7]. While LiDAR offers outstanding accuracy and efficiency, its high cost limits accessibility. In contrast, image-based techniques are affordable, flexible, and capable of producing highly detailed 3D visualizations, leading to their widespread use in industrial inspection and quality control [8,9,10] as well as heritage documentation [11,12,13,14].

Despite their advantages, conventional photogrammetry-based methods struggle with non-collaborative or reflective surfaces, where texture dependence and specular reflections introduce reconstruction noise and inaccuracies [15,16,17]. These challenges underscore the need for advanced techniques capable of handling such complexities. Recently, Neural Radiance Fields (NeRFs) have emerged as a powerful alternative for image-based 3D reconstruction. Introduced by Mildenhall et al. [18] and inspired by earlier attention-based architectures [19], NeRF trains a multilayer perceptron (MLP) to synthesize novel views of a scene through a differentiable rendering process, offering improved realism and fidelity over traditional methods.

**Figure 1:**
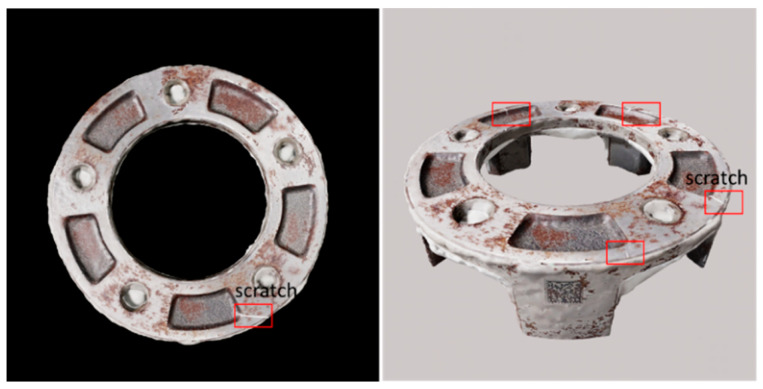
Examples of synthetic defects from top and side view of 3D reconstructed industrial object.

The first step of this work is to identify the most effective NeRF-based methods for generating high-quality 3D reconstructions from our custom industrial datasets, which is critical for a subsequent step 2 to simulate surface defects for the goal of evaluating deep learning-based defect detection systems. To accomplish the first step, utilizing Nerfstudio developed by Tancik et al. [20] and NVlab by NVIDIA [21] to train, evaluate, and visualize the NeRF scenes with good graphic user interface (GUI), we employ a comparative study of various NeRF-based methods, including Instant-NGP [22], Nerfacto [23], Volinga [24], and TensorRF [25]. Our methodology involves collecting three custom datasets of two industrial objects for a 3D reconstruction task, training NeRF models with these custom datasets using Nerfstudio, evaluating the performance of each model, and selecting the most effective NeRF method for generating high-quality 3D reconstructions. Subsequently, the reconstructed industrial 3D object by NeRF method is applied to generate synthetic scratches on their surface, as shown in Figure 1. The synthetic defect images will be used as a test case for deep learning-based models for surface defect detection.

In summary, to address the challenges associated with collecting images of specific defects, our paper proposes a novel unified framework for generating synthetic defects on 3D reconstructed industrial objects. This approach facilitates the creation of high-quality training datasets for deep learning-based object detection models and enables the bridging of the simulation-to-reality gap. The contributions of this work are summarized as follows:

- We created a novel dataset focused on synthetic scratches on 3D industrial object surfaces by combining NeRF-based 3D reconstruction with synthetic defect generation.
- We introduce a framework utilizing NeRF to reconstruct high-fidelity 3D models of industrial objects from 2D images. This approach circumvents the limitations of CAD, particularly licensing restrictions.
- Our framework incorporates the NVIDIA Omniverse Replicator to simulate synthetic scratches on reconstructed 3D objects. This work enables the generation of diverse, annotated datasets critical for training robust deep learning-based defect detectors.
- We benchmark the performance of several YOLO object detectors on our datasets. The experiment’s results demonstrate that incorporating synthetic defects along with real ones in the training process significantly enhances the generalization performance of these models.

## 2. Related Work

Our research focuses on developing a synthetic image generation system for defect detection applications. This system leverages techniques that combine 3D models of individual objects or scenes and rendering pipelines, thereby facilitating the creation of synthetic datasets. Notably, existing datasets typically are created based on CAD models or real-world 3D reconstructions, whereas recent approaches have largely relied on utilizing 3D modeling tools and game engines as rendering frameworks, as illustrated in Table 1. The Normalized Object Coordinate Space (NOCS) dataset [26] comprised real-world background images captured using a tabletop view, paired with objects from ShapeNetCore [27] to generate synthetic images with Unity. The Synthetic HOPE dataset [28] contained 28 scanned daily objects acquired using an EinScan-SE 3D Scanner. To facilitate domain randomization, synthetic images of each object were created under varying levels of occlusion and five distinct lighting conditions utilizing NVIDIA’s Deep Learning Data Synthesizer (NDDS) [29] integrated into Unreal Engine. Additionally, the UOAIS-Sim dataset [30] was generated using the 3D modeling tool BlenderProc [31]. Back et al. utilized this tool to create a dataset of household objects, including bottles and cereal boxes from the KIT Object Database [32], Bigbird [33], and the BOP challenge [34]. These objects were randomly placed on textured surfaces within a predefined scene setup. The SynTable dataset [35] was developed using the NVIDIA Omniverse platform and Isaac Sim Replicator Composer [36]. This dataset consists of tabletop scenes featuring CAD models from the Google Scanned Object dataset [37] and the BOP [34].

Previous studies of synthetic dataset generation provide some insight into our work. However, many aspects need to be customized following our work requirements. Firstly, CAD models are not preferable choices due to licensing and our 3D objects are custom 3D industrial objects without existing 3D datasets. In addition, different from previous works, the rendering pipeline placed 3D objects randomly on scenes following different configurations. In this work, the 3D industrial objects are fixed while the defects are placed randomly on their surfaces. Based on the literature review, the NVIDIA Omniverse Defect Extension, released in March 2023 [38], is selected as the pipeline rendering method, while we aim to further study 3D object reconstruction methods, as presented in Table 2.

Recent advancements in 3D reconstruction using Neural Radiance Fields (NeRFs) have seen widespread application across various domains, each utilizing a range of tailored methods to address specific challenges. In the context of power transmission lines, Zeng et al. [39] employed techniques such as Instant-NGP, Volinga, etc., to handle the complex task of accurately reconstructing these elongated structures from progressive motion sequence images. This approach significantly enhances the precision of the 3D models, particularly in capturing fine details under sparse data conditions. Similarly, heritage conservation has benefited from the work of Mazzacca et al. [40], who utilized a suite of methods including Instant-NGP, Nerfacto, etc., to digitally reconstruct historical artifacts and sites with high fidelity, preserving intricate textures and geometries essential for cultural heritage preservation. For more generalized 3D reconstruction tasks across various datasets, including objects like statues, vehicles, and industrial objects, the authors in [41] have applied methods such as Nerfacto, TensoRF, etc., showcasing the adaptability of NeRF-based techniques to diverse object types and complexities. Caruso et al. in [42] addressed the unique difficulties of reconstructing non-cooperative resident space objects, using Instant-NGP and D-NeRF to accurately model these dynamic and unpredictable objects in space, demonstrating the robustness of these methods in handling challenging scenarios across various domains. Pei et al. in [43] focused on large-scale substation equipment, employing COLMAP [44], NeRF, Instant-NGP, etc., to tackle the challenges posed by industrial environments, including occlusions and variable lighting conditions.

Previous works, particularly those discussed in [41] about industrial objects, have provided valuable insights and approaches for our industrial 3D object reconstruction work, including data collection methods for NeRF-based training, NeRF training processes, and evaluation metrics. However, there are numerous variants of NeRF-based models that require further investigation to narrow down the suitable options. A comprehensive review of the NeRF literature [45] revealed that baseline NeRF models suffer from slow training and inference speeds. Given the computational limitations imposed by our RTX 3080 hardware, we focus on identifying NeRF-based models with fast training times. After consideration, a shortlist of four prominent models emerges, including Instant-NGP, Nerfacto, Volinga, and TensoRF, which will be trained and tested with a unified Nerfstudio framework.

## 3. Proposed Method

**Figure 2:**
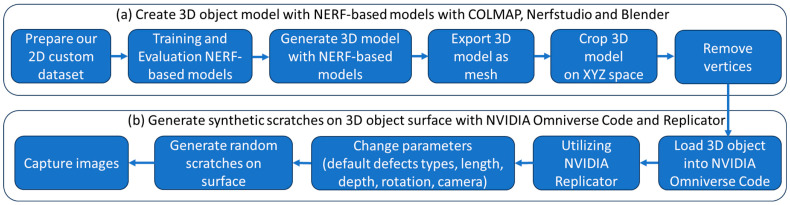
Flow diagram of proposed framework, including (**a**) create 3D reconstructed object based on NeRF models with Nerfstudio and Blender and (**b**) generate synthetic scratches on 3D object surface with NVIDIA Omniverse Code and Replicator.

The proposed method consists of two main parts aimed at creating a comprehensive pipeline for generating 3D object models with synthetic defects, as shown in Figure 2. In the first part, three custom 2D image datasets of two industrial objects are created by capturing images from various angles around these two objects. These images are pre-processed and used to train NeRF-based models to reconstruct the objects’ 3D structure from the 2D data. Then, the reconstructed 3D model is exported as a mesh and further refined by cropping in XYZ space and removing unnecessary vertices for subsequent defect generation. In the second part, the refined 3D model is integrated into NVIDIA Omniverse Code and utilized within the NVIDIA Replicator Defect Extension to generate synthetic defects. Parameters such as defect type, length, depth, and rotation are adjusted, and random scratches are applied to the surface of the object to create diverse defect scenarios. The final step involves capturing images of the 3D model on a different camera setup together with generating labeled annotations specifically for training defect detection tasks. By combining the capabilities of NeRF-based models for 3D object reconstruction and the NVIDIA Replicator for defect generation, our proposed method provides a comprehensive solution for creating synthetic datasets with realistic 3D object models that contain a wide range of surface defects. This approach serves as a powerful tool for developing and testing defect detection models in various industrial and manufacturing contexts. The details of each component are presented as follows.

### 3.1. NeRF-Based Model for 3D Reconstruction

**Figure 3:**
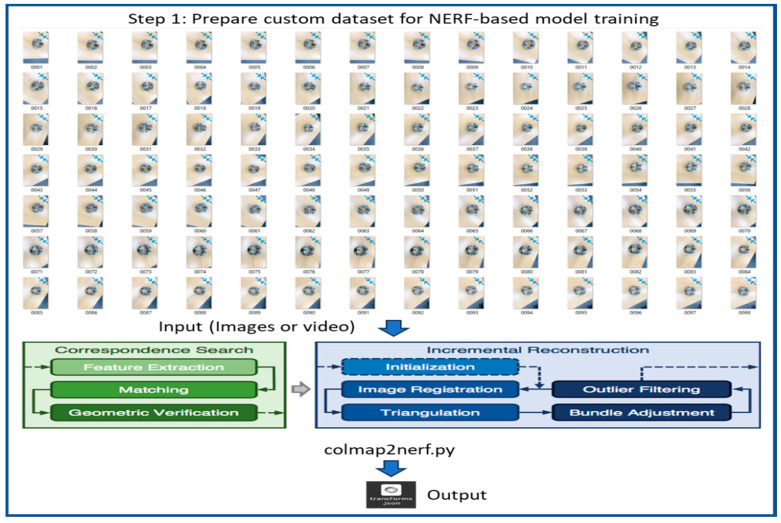
Flow diagram of 3D object reconstruction step 1: Preparation of custom dataset for NeRF training.

The proposed method includes several detailed steps to process the 3D reconstruction of objects, as shown in Figure 3. However, these steps are processed using three main tools or frameworks, including COLMAP, Nerfstudio, and Blender. COLMAP 3.7 is a reliable software tool designed for 3D reconstruction that combines Structure-from-Motion (SfM) and Multi-View Stereo (MVS) algorithms to create accurate 3D models from datasets of 2D images, making it very useful for applications in photogrammetry, computer vision, and 3D reconstruction. In this work, we use COLMAP for camera calibration and feature extraction to prepare our custom dataset for NeRF-based model training. Nerfstudio is an open-source Python-based framework that provides a comprehensive set of tools and APIs for building, training, and deploying NeRF models. This framework offers a flexible and efficient platform for researchers and developers to implement and explore various NeRF architectures, making it a valuable resource for advancing the field of NeRF. Additionally, Nerfstudio integrates with the real-time web viewer, providing an interactive experience for users to visualize and explore their generated scenes, thereby simplifying the process of training, testing, and interacting with reconstructed 3D objects and scenes. By only utilizing the Nerfstudio framework for all four NeRF models’ training and testing, we streamline the workload of setting up Python 3.10 environments for training different NeRF models. Blender is a comprehensive set of tools, including modeling, animation, rendering, physics, and scripting capabilities, making it an ideal platform for refining and improving the accuracy of reconstructed models created using photogrammetry or NeRF. The plugin installations in Nerfstudio for NeRF and Meshroom [46] for photogrammetry further enhance its versatility.

**Figure 4:**
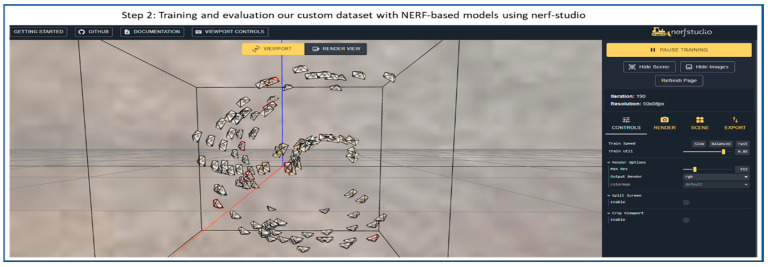
Flow diagram of 3D object reconstruction step 2: Training and evaluation of NeRF models using Nerfstudio.

**Figure 5:**
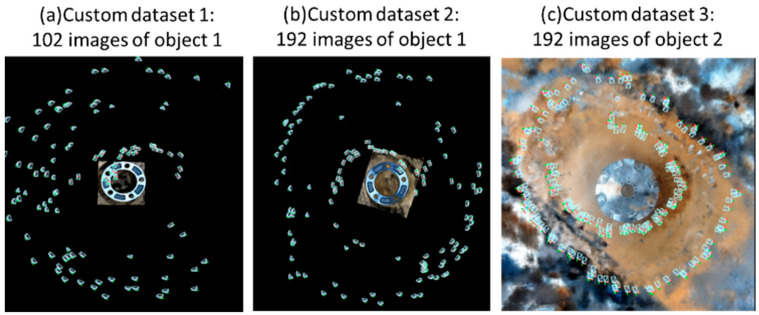
Example of custom dataset for training NERF-based models for 3D object reconstruction.

**Figure 6:**
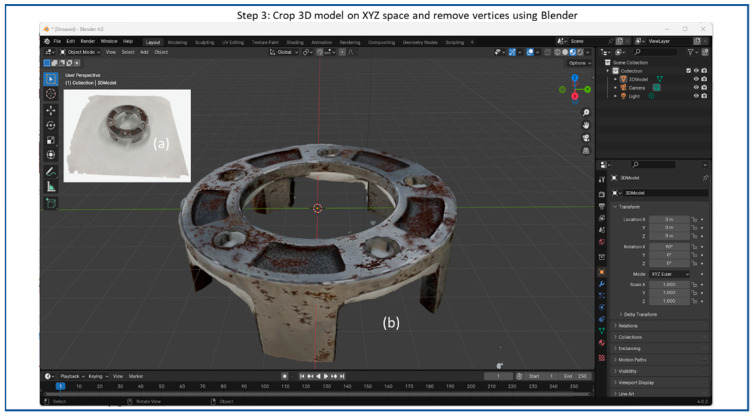
Flow diagram of 3D object reconstruction step 3: Blender for refining 3D reconstructed object including (**a**) original NeRF-reconstructed 3D model and (**b**) refined 3D model cropping XYZ spaces.

**Figure 7:**
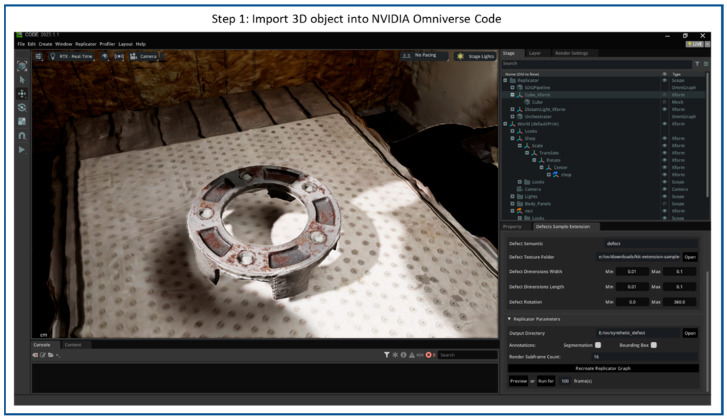
Flow diagram of synthetic scratches generation step 1: Import 3D reconstructed object into NVIDIA Omniverse Code.

The flow diagram of our 3D object reconstruction mechanism is presented in Figure 3, Figure 4, Figure 5, Figure 6 and Figure 7. The first step is the preparation of a custom dataset for NeRF training, which is shown in Figure 3. Initially, a custom 2D image dataset of the target object is prepared by capturing images using an iPhone camera, followed by preprocessing using COLMAP to generate camera poses and sparse point clouds, which are then exported to a “.json” file for subsequent NERF-based model training and evaluation. The training of NeRF models necessitates the availability of initial camera parameters in a format compatible with the original NeRF codebase [47], which requires camera data to be stored in a “.json” file. To facilitate this process, a Python script called “colmap2nerf” utilizes COLMAP to extract essential camera data from video files or sequences of images.

Figure 4 illustrates step 2, in which the Nerfstudio interface is used for training and evaluating NeRF models. It displays a 3D visualization of camera poses surrounding the reconstructed object. These camera positions indicate viewpoints captured during dataset preparation, which are used to optimize the NeRF’s volumetric representation. The right panel provides training controls and rendering options, allowing real-time monitoring and parameter adjustments during model training.

We will briefly introduce and present the outstanding perspective of the four NeRF models that we chose as competitors for training our custom dataset with Nerfstudio. Instant-NGP stands out as a promising approach due to its utilization of multi-resolution hashed positional encoding as an additional learned feature. This enables accurate scene representation using compact and efficient MLPs, resulting in extremely fast training and inference times. Notably, the authors implemented Instant-NGP to run entirely on a single CUDA kernel. In contrast, TensoRF considers the full volume field as a 4D tensor and proposes to factorize the tensor into multiple compact low-rank tensor components for efficient scene modeling. Experiments demonstrate that TensoRF with Candecomp/Parafac (CP) decomposition achieves fast reconstruction with improved rendering quality and a smaller model size compared to NeRF. Furthermore, TensoRF with Vector–Matrix (VM) decomposition enhances rendering quality while reducing reconstruction time and maintaining a compact model size. A key advantage of TensoRF is its standard PyTorch implementation, which provides efficiency gains without requiring customized CUDA kernels like Instant-NGP. Nerfacto builds upon Instant-NGP by using both the fully fused MLP and hash encoder, but with some differences in learning rate schedulers, hyperparameters for sampling, and camera gradient calculation. These modifications aim to improve the performance and stability of the model. Finally, Volinga is a modification of Nerfacto that allows conversion to NVOL files, which is a specialized format optimized for fast and efficient storage to be easily integrated into Unreal Engine.

It is crucial to note that the training process can be highly sensitive to the quality and consistency of the dataset. Specifically, datasets should possess good coverage, be free from mislabeled camera data, and be without blurry frames caused by motion blur or defocus blur. Furthermore, incorrect scale or offset in the camera positions, insufficient number of images, and COLMAP’s inability to extract camera data are common issues that can significantly impact the performance of NeRF models. In the experiment setup, we conduct experiments to verify the importance of good coverage and the number of images. To train NeRF-based models for 3D object reconstruction, three custom datasets of two sample industrial objects (object 1 and object 2) by [48] are utilized, as seen in Figure 5. To evaluate the contribution of the coverage of images to the accuracy of reconstruction, we use custom dataset 1 of 102 images and custom 2 of 192 images of the same object 1. As shown in Figure 5a, there is missing information on the right side of object 1 as compared to that in Figure 5b.

To test the generalization performance of NeRF-based models, we use two samples, object 1 and object 2, with the same number of 192 images to train the four models with the Nerfstudio framework, including Instant-NGP, Nerfacto, Volinga, and TensoRF. For all experiments, the number of training iterations is set as 30 thousand iterations with the default settings configuration of each model based on the Nerfstudio framework.

Subsequently, the resulting trained 3D object model is then exported as a mesh file format, such as “.obj” or “.stl” or point cloud, for further processing in step 3. Figure 6 shows the Blender interface displaying a 3D reconstructed model, including (a) the original NeRF-reconstructed 3D model from step 2, and (b) the refined 3D model by cropping XYZ spaces. In step 3, optionally the XYZ space of the 3D model can be cropped to focus on specific regions of interest, and unwanted vertices can be removed to refine the model’s appearance or reduce complexity.

### 3.2. Synthetic Surface Defect Generation

NVIDIA Omniverse Code is a powerful platform designed for building and customizing real-time simulations and virtual environments. This platform gives developers a flexible and scalable framework for building advanced 3D workflows, allowing them to bring together various tools and applications within one unified system. Omniverse Code’s expandable architecture also supports custom extensions, making it work well for many different specialized uses, from procedural content creation to AI-powered automation. One extension within Omniverse Code is the Defect Sample Extension [38], which is specifically designed for creating synthetic data by adding defect textures to 3D models, also called Prims. This extension works particularly well in situations where defects like scratches or wear marks need to be simulated on objects for jobs like training deep learning models or visual inspections. The Defect Sample Extension uses Omniverse’s Replicator functionality, a powerful tool for randomization and synthetic data creation.

**Figure 8:**
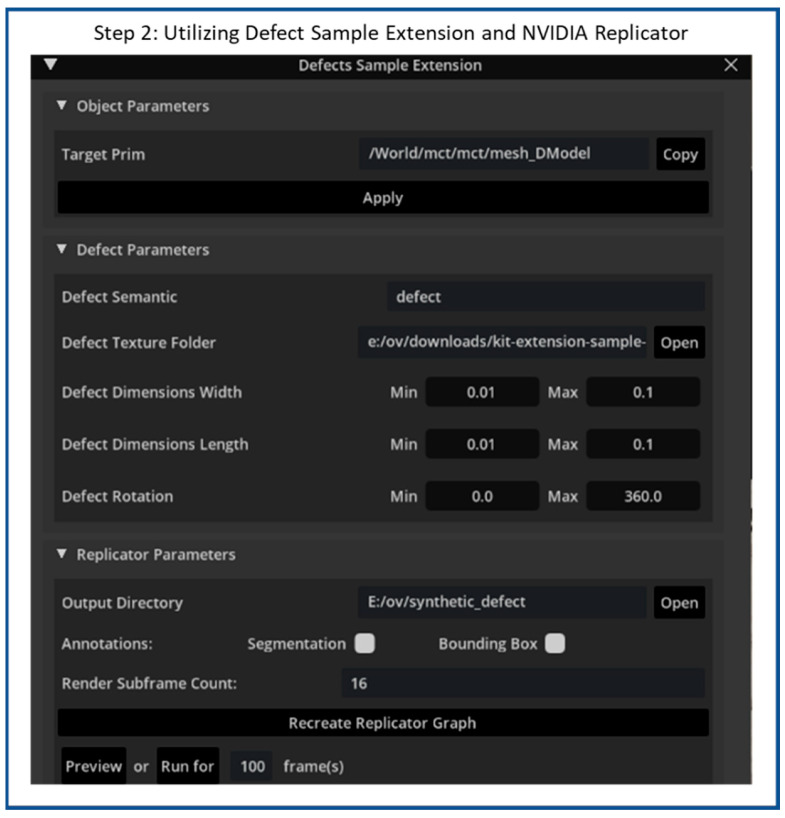
Flow diagram of synthetic scratches generation step 2: Utilize Defect Sample Extension and replicator.

**Figure 9:**
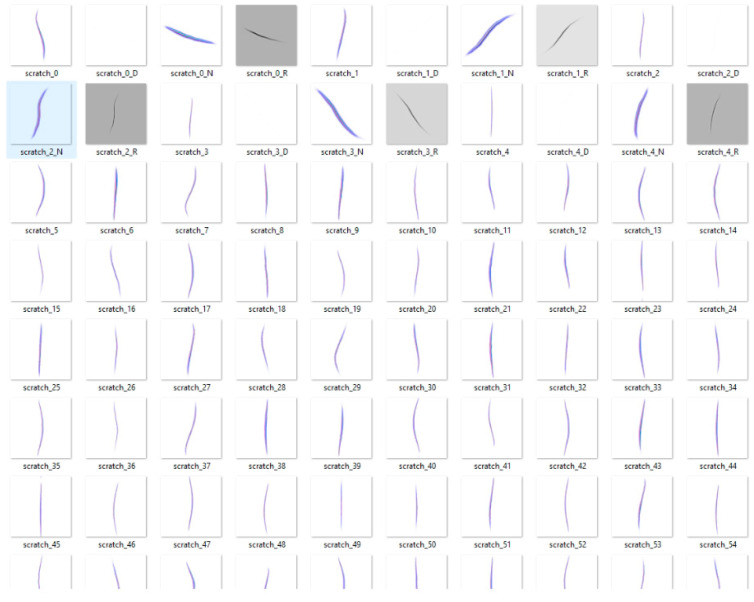
Example of 100 scratch defect texture samples.

**Figure 10:**
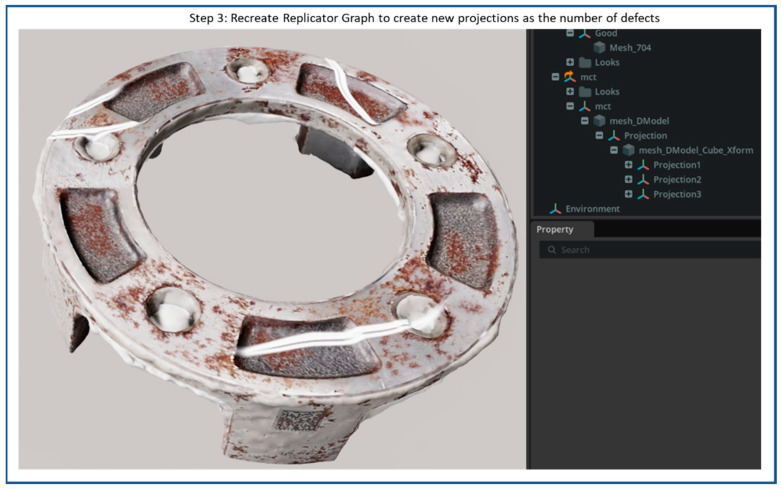
Flow diagram of synthetic scratch generation step 3: Recreate replicator graph to create new projections as additional defects.

**Figure 11:**
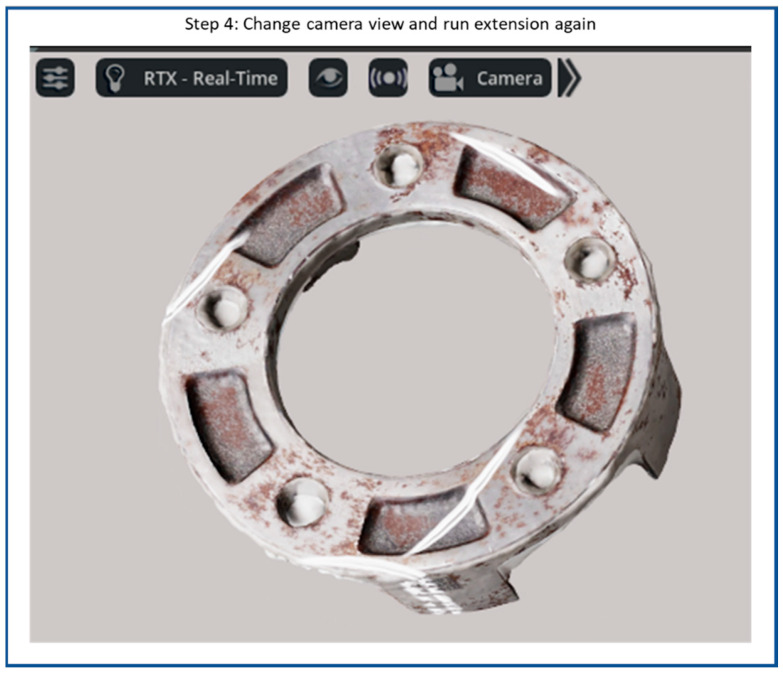
Flow diagram of synthetic scratch generation step 4: Change camera view and capture images.

The processes of creating synthetic scratches using NVIDIA Omniverse Code and the Defect Sample Extension are shown in Figure 7, Figure 8, Figure 9, Figure 10 and Figure 11, including four steps. The workflow starts by importing a 3D reconstructed object into the Omniverse Code as step 1, which acts as the base model for defect replicators. Figure 7 illustrates the NVIDIA Omniverse Code interface displaying a 3D reconstructed industrial object placed at the center of the workspace. The camera is positioned above the object in a slightly oblique top-down view, while RTX real-time lighting enhances material realism, emphasizing surface roughness and metallic reflections for accurate synthetic defect rendering.

Next, the Defect Sample Extension is used to project a selected defect texture onto a target Prim, as in step 2. Figure 8 illustrates the Defect Sample Extension interface within the NVIDIA Omniverse Code environment, which provides a configurable tool for generating and controlling the synthetic surface defect replicator on 3D models. The interface is organized into three major sections—Object Parameters, Defect Parameters, and Replicator Parameters—each serving a specific role in the defect replicator workflow.

In the Object Parameters section, the user specifies the target primitive (Prim) on which the defect texture will be projected. In this case, the target Prim is imported 3D industrial object used for synthetic defect generation. This step establishes the link between the selected mesh and the subsequent defect projection process. The Defect Parameters section allows users to control the visual and spatial characteristics of the synthetic defects. Parameters such as the Defect Semantic, Defect Texture Folder, and Defect Dimensions (width, length, and rotation) are provided to define the appearance, scale, and orientation of each defect. Figure 9 illustrates a sample set of 100 synthetic scratch textures that are loaded into the extension via the Defect Texture Folder parameter.

By setting minimum and maximum values, users can introduce variability, enabling the generation of defects with randomized sizes and orientations to enhance dataset diversity. Finally, the Replicator Parameters section defines how the synthetic data will be produced and stored. The user can set an output directory for saving rendered images and metadata, as well as enable options for generating segmentation masks or bounding-box annotations. The extension also includes controls for rendering frame count (or capturing each image per frame) according to the specified defined parameters. By doing so, thousands of synthetic images and corresponding metadata can be generated, making it ideal for creating datasets for training AI models in tasks such as defect detection or quality inspection.

Figure 10 illustrates the customized multi-defect generation process implemented within the NVIDIA Omniverse Code. In this configuration, our work extends the standard Defect Sample Extension to allow multiple defect projections on a single target Prim, thereby creating more complex and realistic surface conditions. Each projection node shown in the scene hierarchy (Projection, Projection 1, Projection 2, and Projection 3) corresponds to four individual synthetic defects applied to the 3D model’s surface. By defining multiple projection nodes, users can simulate various defects across different regions of the same object.

Figure 11 presents the final step 4 of the synthetic defect generation process in the NVIDIA Omniverse Code. In this step, the scene configuration has been refined by adjusting the object’s spatial orientation, camera angle, and lighting direction to achieve diverse visual perspectives. These modifications allow users to regenerate the scene and replicate multiple frames with variations in viewpoint and illumination. By capturing the same object under different conditions, the workflow produces a richer and more comprehensive synthetic dataset, which is particularly valuable for training AI models to detect surface defects under varying lighting and viewing environments. Finally, the labeled images consist of 1890 original synthetic images, and the corresponding annotation xml files of the “scratch” class.

To verify the effectiveness of synthetic defect generation, we conduct experiments of defect detection ablation in cases with and without our synthetic defects on the NEU-DET dataset. In this work, to benchmark several You-Only-Look-Once (YOLO) models, including YOLOv5 [49], YOLOv6 [50], YOLOX [51], YOLOv7 [52], YOLOv8 [53], and YOLOv10 [54] for the task of scratch detection, our synthetic dataset of 1890 images is split into 80/20 percentages of training and validation sets, as can be seen in Table 3. It is noted that we omit YOLOv9 [55] due to the constraint of computational resources with RTX 3080 for training YOLOv9. In addition, together with our synthetic dataset, we utilize a combination of the test case with real “scratch” data from the NEU-DET dataset of 300 images [56] and our synthetic “scratch” to verify the effectiveness of using the synthetic dataset for enhancing the accuracy of detection models. For all experiments, the input image size is 640 × 640, the batch size is 16, and the epochs are 30 epochs with default hyperparameters.

## 4. Experiment Setup and Results

### 4.1. NeRF-Based 3D Reconstruction Evaluation Metrics

Three image quality assessments including the Peak-Signal-to-Noise Ratio (PSNR), Structural Similarity Index (SSIM) [57], and Learned Perceptual Image Patch Similarity Index (LPIPS) [58] are evaluated in this work, where higher values of PSNR and SSIM indicate better image quality and structural accuracy, respectively, and lower LPIPS values indicate lower perceptual error and better visual fidelity.

PSNR is an indicator used to evaluate the amount of image quality loss, as the higher PSNR means less loss and better quality. PSNR is particularly useful for assessing the sharpness and clarity of NeRF-rendered images.

(1) $$PSNR=10\cdot {log}_{10}\left(\frac{{MAX}_{I}^{2}}{MSE}\right)$$

(2) $$MSE=\frac{\sum_{M,N}{\left[I_{1}\left(m,n\right)-I_{2}\left(m,n\right)\right]}^{2}}{M*N}\;$$

where M and N mean the width and the height of the image, I(m,n) means the color value of the m,n coordinates, and MAX represents the amount of information per pixel (RGB:255 × 3).

SSMI measures the similarity between two images (x, y) based on their structural content, luminance, and contrast. Higher SSIM values denote better quality, capturing both luminance and structural fidelity between rendered and ground-truth images. In NeRF evaluation, SSIM helps to understand how well the NeRF-based model preserves essential features and spatial patterns.

(3) $$SSMI\left(x,y\right)={\left[l(x,y)\right]}^{\alpha }\cdot {\left[c(x,y)\right]}^{\beta }\cdot {\left[s(x,y)\right]}^{\gamma }$$

(4) $$l\left(x,y\right)=\frac{2\mu_{x}\mu_{y}+C_{1}}{\mu_{x}^{2}+\mu_{y}^{2}+C_{1}}$$

(5) $$c\left(x,y\right)=\frac{2\sigma_{x}\sigma_{y}+C_{2}}{\sigma_{x}^{2}+\sigma_{y}^{2}+C_{2}}$$

(6) $$s\left(x,y\right)=\frac{\sigma_{xy}+C_{3}}{\sigma_{x}\sigma_{y}+C_{3}}$$

(7) $$\mu_{x}=\frac{1}{N}\sum_{i=1}^{N}x_{i}$$

(8) $$\sigma_{x}={\left(\frac{1}{N-1}\sum_{i=1}^{N}{\left(x_{i}-\mu_{x}\right)}^{2}\right)}^{1/2}$$

(9) $$\sigma_{xy}=\frac{1}{N-1}\sum_{i=1}^{N}\left(x_{i}-\mu_{x}\right)\left(y_{i}-\mu_{y}\right)$$

where α=β=γ=1 as the luminance, contrast, and structure of the image, and $\mu_{x}$, $\mu_{y}$, $\sigma_{x}$, $\sigma_{y},$ $\sigma_{xy}$ as mean intensities, standard deviations, and covariance of the images x and y.

LPIPS is a perceptual metric that measures the similarities between images based on deep features extracted from a trained NeRF-based network. Lower LPIPS values indicate better perceptual similarity, meaning that the NeRF-rendered image is closer to the ground-truth image in terms of human perception.

(10) $$LPIPS(x,y)=\sum_{l}\frac{1}{H_{l}W_{l}}\sum_{h,w}{\left\|w_{l}⊙\left({\hat{y}}_{h,w}^{l}-{\hat{x}}_{h,w}^{l}\right)\right\|}_{2}^{2}$$

where ${\hat{x}}^{l}$ and ${\hat{y}}^{l}$ are the normalized feature maps of the images x and y at layer one of the network, $H_{l}$ and $W_{l}$ are the dimensions of the feature maps, and $w_{l}=1\forall l$ is equivalent to computing cosine distance.

### 4.2. YOLO-Based Object Detection Evaluation Metrics

For this work, we have conducted experiments on the NVIDIA GeForce RTX3080 hardware platform PyTorch framework and Python language. Other configurations are CUDA 11.2, Cudnn 8.1, and PyTorch 1.12.1. To evaluate the performance of object detection tasks, the following metrics are used, including precision, recall, precision and recall curve, F1-confidence score, and mAP (mean Average Precision) [59].

(11) $$Precision=\frac{TP}{TP+FP}$$

(12) $$Recall=\frac{TP}{TP+FN}$$

(13) $$F1=2\times \frac{Precision\times Recall}{Precision+Recall}$$

(14) $$AP=\int_{0}^{1}P(R)dR$$

(15) $$mAP=\frac{1}{N}\int_{0}^{1}P\left(R\right)dR$$

where TP is true positive (correctly predicted objects), FP is false positive (incorrectly predicted objects), FN is false negative (undetected objects), AP is average precision (the area under the precision–recall curve for each class), and mAP is the mean of AP values across all classes N.

In this work, there is only one class “scratch” as N = 1. A higher mAP indicates better overall performance in detecting and correctly classifying objects across all classes in the dataset. Model sizes of params and computation complexity FLOPs are chosen to compare the computational cost of different models.

### 4.3. Experiment Results

#### 4.3.1. 3D Reconstruction Results

To assess the impact of coverage on 3D object reconstruction accuracy, we compare two custom datasets of the same object 1, including dataset 1 with 102 images and dataset 2 with 192 images. The primary difference between these two datasets lies in the volume of image overlap. The increased overlap in dataset 2 is crucial for achieving higher accuracy in 3D reconstruction. For instance, when trained on dataset 2 with 192 images, all models show improved results, with Instant-NGP achieving a higher PSNR of 22.83 and an SSIM of 0.85 and a lower LPIPS of 0.46, compared to its performance on dataset 1 with 102 images, where it achieved PSNR of 18.90, SSIM of 0.78, and LPIPS of 0.57. Similarly, other models like Nerfacto and Volinga also demonstrated significant improvements in PSNR and SSIM and reduction in LPIPS when trained on dataset 2. These findings underscore the importance of coverage, as the increased overlap in dataset 2 contributed to more accurate and complete 3D reconstructions across all evaluated models.

To evaluate the generalization performance, four models are trained on two datasets, namely dataset 2 and dataset 3 as shown in Table 4. Each dataset contains 192 images of two different objects (object 1 and object 2), which are split into training and validation sets of 173 and 19 images, respectively. The quantitative results indicate that Nerfacto achieves a consistent performance with high PSNR, high SSIM, and low LPIPS across both datasets, demonstrating robust generalization across different object configurations. Instant-NGP, while showing the best performance on dataset 2 with a PSNR of 22.83, exhibits a significant performance drop on dataset 3, indicating a potential sensitivity to variations in object features or viewpoints. Volinga maintains moderate performance, with slight variations between the datasets, indicating that it captures structural details well but struggles with reducing perceptual errors. TensoRF, on the other hand, shows the weakest generalization, with consistently lower PSNR and higher LPIPS values, indicating challenges in maintaining image quality and perceptual accuracy across diverse datasets. These results emphasize that Nerfacto is the most reliable model for achieving high-quality, perceptually accurate reconstructions, while Instant-NGP and Volinga may require further refinement. TensoRF’s performance highlights a need for optimization to improve its generalization ability across varied datasets.

The number of images for training NeRF datasets are quite crucial because it is regular to see large discrepancies when using a small number of image datasets. However, in our work, the goal of 3D reconstructed objects is only used to render synthetic defects on their surface. Compared to applications like heritage preservation, the details and precise texture are much more needed than in our work. Although more realism compared to the real object would be much better, we need to consider the relationship between the training time and affordable detailed 3D reconstructed objects.

#### 4.3.2. Synthetic Defect Rendering Results

**Figure 12:**
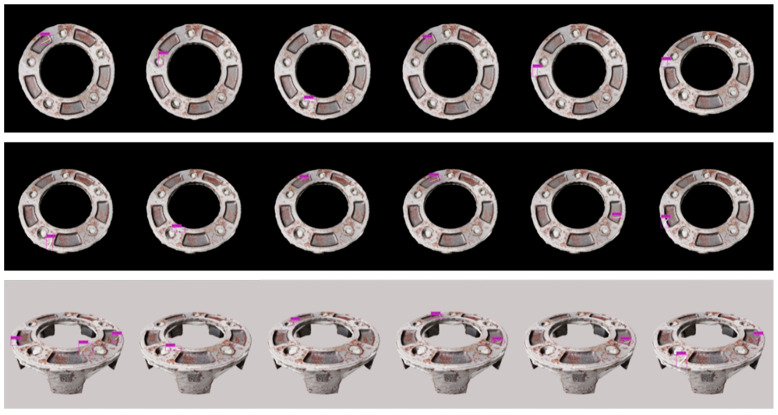
Example of synthetic defect rendering results on different settings.

Figure 12 illustrates an example of synthetic rendering results at different iterations. In each iteration, various parameters, such as defect settings, number of projections, background scene, lighting conditions, and 3D object orientation, are reconfigured and re-executed to generate new outputs. The original rendered image did not include visible bounding boxes; instead, the annotation data were stored in the corresponding metadata files. For better visualization and clarity, bounding boxes have been added to this image to highlight the defect regions. To mimic the NEU-DET dataset, most renderings are captured from a top-down camera angle with a black-and-white background, ensuring consistency with common industrial inspection viewpoints. However, additional configurations were also introduced to increase variation, including slightly tilted camera angles and wider object perspectives. Notably, the synthetic defects were designed to appear narrow, elongated, and diverse in both depth and direction, exhibiting various shapes such as straight, curved, and diagonal scratches across the surface. This diversity in defect geometry and orientation enhances the realism of the dataset and provides a richer foundation for training defect detection models.

**Figure 13:**
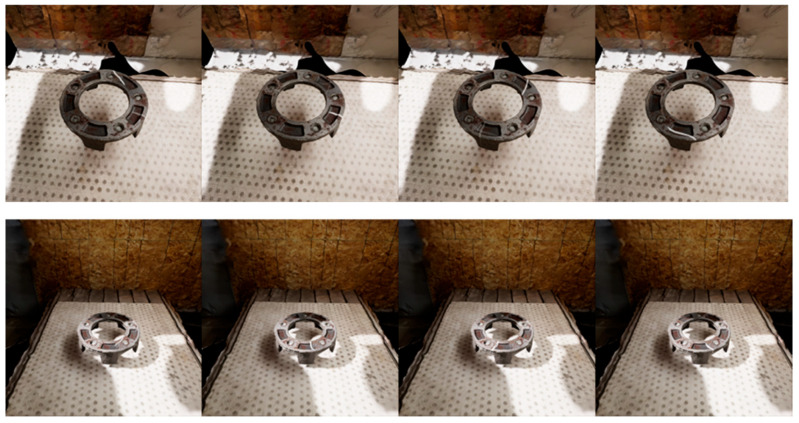
Example of synthetic defect rendering results on different illumination settings.

Figure 13 demonstrates a series of synthetic renderings showcasing the impact of variable illumination and surface secularity on a reconstructed metallic component. Each image depicts the same object under different lighting intensities, incident angles, and shadow directions, simulating diverse real-world industrial lighting conditions. The variations in brightness and reflection patterns across the sequence highlight the surface secularity typical of metallic materials, where light reflects unevenly depending on the viewing angle and surface roughness. By incorporating such variations, the synthetic dataset effectively captures a wide range of lighting scenarios, including strong direct light, diffuse illumination, and mixed-shadow environments. This diversity ensures that machine vision models trained on the dataset gain improved robustness and generalization when deployed in real industrial settings with uncontrolled or fluctuating illumination conditions.

#### 4.3.3. Deep Learning Object Detection Models Results

The quantitative evaluation of various deep learning object detection models, as presented in Table 5, demonstrates significant differences in evaluation metrics, particularly in terms of mAP@0.5, precision, recall, and F1-Score. Among the models evaluated, YOLOv8-s stands out with the highest performance, achieving a mAP@0.5 of 96.4 percent and mAP@0.5:0.95 of 53 percent. The model also exhibits a high precision of 96.3 percent and a recall of 92 percent, resulting in a superior F1-Score of 94 percent. These results indicate that YOLOv8-s offers exceptional accuracy in our custom defect detection, ensuring reliable and consistent performance across different sizes and shapes of defects.

Similarly, YOLOv8-n shows strong performance with a mAP@0.5 of 92.7 percent and mAP@0.5:0.95 of 49.5 percent, along with a high F1-Score of 89 percent. The YOLOv6 series, particularly the YOLOv6-s model, shows the second-best overall performance among the models compared. YOLOv6-s reaches a mAP@0.5 of 91.3 percent, mAP@0.5:0.95 of 41.2 percent, precision of 92.4 percent, recall of 89.1 percent, and an F1-Score of 90.7 percent, placing it just behind YOLOv8-s in terms of accuracy and reliability. This model effectively balances high accuracy with manageable computational cost, having 18.05 million parameters and 45.17 giga FLOPs, making it suitable for applications requiring strong detection capabilities. YOLOv6-n, with its mAP@0.5 of 87.2 percent, precision of 88.8 percent, recall of 83.2 percent, and F1-Score of 85.9 percent, also provides strong performance, especially considering its lower computational demands. Overall, the YOLOv6 models are excellent choices for tasks that need high precision and recall while maintaining reasonable computational efficiency.

The YOLOX models, particularly YOLOX-s, show the third-best performance in terms of mAP, though they lack comprehensive data for precision, recall, and F1-Score, making it difficult to fully evaluate their overall effectiveness. YOLOX-s reaches a mAP@0.5 of 90.6 percent and a mAP@0.5:0.95 of 48.42 percent, which are strong indicators of its accuracy in our custom defect detection. YOLOX-n, with a mAP@0.5 of 86.97 percent and mAP@0.5:0.95 of 40.59 percent, also performs well, especially considering its extremely low computational complexity of 0.91 million parameters and 1.08 giga FLOPs.

The YOLOv10 models, as the latest variants in the YOLO family, introduce notable advancements in architecture and optimization that slightly reduce the computational complexity compared to YOLOv8. YOLOv10-s achieves a mAP@0.5 of 89.4 percent and mAP@0.5:0.95 of 48.5 percent, with precision at 86.6 percent, recall at 82 percent, and an F1-Score of 84 percent. These metrics suggest a well-balanced model with solid performance and computational efficiency, featuring 8.03M parameters and 24.4G FLOPs. Similarly, YOLOv10-n offers a mAP@0.5 of 81.6 percent and mAP@0.5:0.95 of 40.2 percent, with precision at 80.2 percent, recall at 73.1 percent, and an F1-Score of 76 percent, making it a lightweight option for scenarios where lower computational complexity is crucial. However, despite these improvements, the YOLOv10 models exhibit a slight downgrade in performance when applied to our custom dataset compared to earlier models like YOLOv8-s, YOLOv6-s, and even YOLOX. While YOLOv10-s and YOLOv10-n still perform reasonably well, they fall short in achieving the same level of accuracy and reliability as their predecessors on this specific dataset. This suggests that while YOLOv10 variants bring the latest innovations, their effectiveness may vary depending on the dataset and specific application, highlighting the importance of model selection based on the context of use.

The YOLOv5 models can be considered as a baseline for latter YOLO variants, and still have better performance than YOLOv7. YOLOv5-n, with its mAP@0.5 of 70.4 percent and mAP@0.5:0.95 of 30.7 percent, delivers an adequate precision of 73.2 percent, recall of 63.7 percent, and F1-Score of 69 percent while maintaining a lightweight computational profile with 1.76 million parameters and 4.1 giga FLOPs. YOLOv5-s improves upon this with a mAP@0.5 of 85.1 percent and mAP@0.5:0.95 of 41 percent, offering a higher precision of 84.2 percent, recall of 80.3 percent, and F1-Score of 82 percent, along with 7.01 million parameters and 15.8 giga FLOPs.

On the lowest end of the performance, models like YOLOv7 and YOLOv7-Tiny demonstrate significantly lower mAP and F1-Scores, suggesting limitations in their ability to detect objects with high accuracy. The significant reduction in precision and recall of YOLOv7 further indicates potential challenges in maintaining consistent detection performance.

Overall, the experimental results highlight that models with higher mAP values generally offer better accuracy in object detection tasks. Additionally, a small bandgap between precision and recall, coupled with a high F1-Score, is indicative of a model’s balanced and reliable performance. Among the models tested, YOLOv8-s emerges as the top performer, offering the best trade-off between accuracy, precision, and computational efficiency, making it the most suitable choice for our custom defect detection task.

The incorporation of synthetic data into the training process of deep learning object detection models yields significant improvements in generalization performance, as demonstrated by the analysis presented in Table 6. Models that are trained on both real and synthetic data exhibit higher mAP scores, enhance precision and recall, and improve F1-Scores compared to those trained solely on real data. When trained exclusively on real data, models such as YOLOv8-s and YOLOv6-s show strong performance, with mAP@0.5 scores of 73.5 percent and 77.4 percent, respectively. However, integrating synthetic data into the training process leads to a notable improvement in generalization performance across most models. For instance, YOLOv6-s, which initially achieved a mAP@0.5 of 77.4 percent when trained on real data alone, sees an increase to 81.9 percent mAP@0.5 when synthetic data are included. Similarly, its mAP@0.5:0.95 improves from 41.6 percent to 45.1 percent, demonstrating enhanced detection accuracy across various IoU thresholds. This improvement is further reflected in the F1-Score, which remains high at 76.2 percent, indicating a balanced and reliable detection capability.

YOLOv8-s also benefits significantly from the cooperation training with synthetic data, with its mAP@0.5 rising from 73.5 percent to 75 percent and mAP@0.5:0.95 improving from 33.3 percent to 36.2 percent. The corresponding increase in precision and recall underscores the model’s improved ability to generalize to unseen real-world scenarios, which is crucial for robust object detection. Smaller models like YOLOv6-n and YOLOv10-n also show noticeable gains when synthetic data are included in the training. YOLOv6-n’s mAP@0.5 jumps from 61.7 percent to 80.5 percent, with a significant boost in mAP@0.5:0.95 from 28.3 percent to 41.6 percent. This model’s F1-Score increases from 60.2 percent to 76 percent, illustrating a marked improvement in detection performance and generalization. Similarly, YOLOv10-n experiences an increase in mAP@0.5 from 36.7 percent to 49.3 percent and an F1-Score improvement from 44.6 percent to 49 percent.

The results clearly demonstrate the effectiveness of incorporating synthetic data into the training process. Across all evaluation metrics, models trained on both real and synthetic data consistently outperform those trained on real data alone. The synthetic data appear to provide additional variance in the training set, which helps the models generalize better to new, unseen real-world scenarios. This is particularly evident in the higher mAP scores and more balanced precision and recall, leading to improved F1-Scores. Overall, the integration of synthetic data serves as a valuable strategy to boost the performance and robustness of object detection models, enhancing their applicability in diverse and dynamic environments.

**Figure 14:**
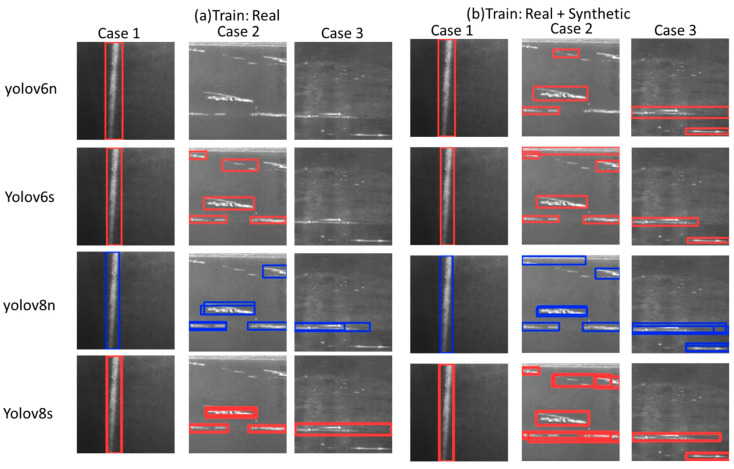
Qualitative comparison of (**a**) training with only real defects, and (**b**) training with both real and synthetic defects in three cases.

In addition to the quantitative improvements observed, a qualitative analysis of the top-performing YOLO-family models, YOLOv8 and YOLOv6, verify the substantial impact of combining real and synthetic data during training on their ability to detect and localize small defects. Figure 14 presents a qualitative comparison of (a) training with only real defects, and (b) training with both real and synthetic defects in three cases, highlighting how these models perform in accurately identifying and localizing various defects in sizes and shapes that are often challenging to detect. In case 1, there is only a big vertical scratch compared to a variety of small horizontal scratches across the images, as in case 2 and case 3. It is seen that YOLOv6 models fail behind YOLOv8 in the case of detecting small scratches in case 2 and case 3 while training only with real defects, with only YOLOv6s able to detect small scratches in case 2. Even though YOLOv8 models successfully detect small defects in both cases 1 and 2, these models suffer from multiple localizing bounding boxes of small scratches with many misdetections. YOLOv6 effectively balances detection accuracy and localization precision. The precise bounding boxes generated by YOLOv6 are tightly aligned with defect boundaries, highlighting the model’s reliability.

Overall, the qualitative results clearly follow the trend observed in the quantitative analysis. Models trained on a combination of real and synthetic data exhibit superior generalization performance, particularly in challenging scenarios involving small defect detection. The ability of YOLOv8 and YOLOv6 to accurately identify and localize small defects, even in previously unseen conditions, underscores the value of incorporating synthetic data into the training process. This approach not only improves the robustness of these models but also ensures that they maintain high precision and accuracy in practical applications where detecting fine details is crucial.

## 5. Conclusions

This study successfully demonstrates a comprehensive framework integrating 3D industrial object reconstruction with synthetic defect generation to enhance digital twin and smart factory applications. By leveraging NeRF-based techniques, specifically Instant-NGP and Nerfacto, we effectively reconstructed high-quality 3D models of industrial objects from smartphone-captured videos. Our comparison of various NeRF-based models revealed Instant-NGP and Nerfacto as superior in generating detailed and accurate 3D reconstructions. However, we compared NeRF methods on two datasets with a limited number of images, which may have contributed to variations in reconstruction quality and may not fully reflect the generalization capability of the NeRF models. In future work, it will be essential to conduct experiments on more diverse industrial object datasets to better evaluate model robustness under realistic conditions. In addition, our proposed framework can be readily extended to other industrial objects with limited additional effort, as we have already established an effective workflow for dataset preparation and a flexible development environment. This allows for efficient adaptation of the framework to new industrial object types, making it suitable for broader applications in industrial 3D reconstruction tasks.

Following the reconstruction, the 3D models were refined to generate synthetic defects on their surfaces using NVIDIA Omniverse Replicator, demonstrating the feasibility of creating a diverse dataset. The evaluation of YOLO-based object detection models on both cases of training with real and real-plus-synthetic defect datasets indicates that training with a combination of real and synthetic defects significantly improves model performance. Specifically, YOLOv6n showed an 18.8 percent enhancement in mAP@0.5, while YOLOv8s demonstrated a 1.5 percent improvement, highlighting the potential of synthetic data to boost detection capabilities. This improvement underscores the effectiveness of synthetic defects in enhancing the generalization of deep learning models in defect detection tasks.

This work has several limitations that should be addressed to further enhance its impact. One notable limitation is the focus on a single popular type of defect, namely a scratch. The current research primarily deals with one defect type, which restricts the generalizability of the synthetic defect generation and its application across various industrial scenarios. To overcome this limitation, future work will include a broader range of defect types, thereby creating a more comprehensive dataset that can better simulate real-world conditions and improve the robustness of the defect detection models.

Another area for improvement lies in the 3D reconstruction techniques used in the study. Although Instant-NGP and Nerfacto demonstrated effective performance in generating high-quality 3D models, there is potential for further advancements. Exploring alternative reconstruction methods, such as Gaussian splatting or other emerging techniques, could enhance the accuracy and detail of the 3D models.

Additionally, while YOLO-based models showed promising results, incorporating other object detection frameworks such as RTMDet could offer further performance improvements. RTMDet, known for its robust detection capabilities, might provide enhancements in detecting defects by leveraging its unique architecture and training approaches. Future research should explore the integration of RTMDet alongside YOLO models and other deep learning frameworks to improve defect detection performance.

## Figures and Tables

**Table 1:** Related work of synthetic data generation for object detection task.

Reference	Object	Methods
SynTable 2023 [35]	CAD/YCB	NVIDIA Omniverse Issac Sim
UOAIS-SIM 2023 [31]	3D Assets	Blender
Synthetic HOPE 2021 [28]	Daily Object	NDDS Unreal
NOCS 2019 [26]	ShapeNetCore	Unity

**Table 2:** Related work of NeRF-based 3D reconstruction.

Reference	Object	Methods	Evaluation Metrics
2023 [39]	Power Transmission Line	Instant-NGP Volinga	PSNR SSIM LIPPS fps
2023 [40]	Heritage	Instant-NGP Nerfacto TensoRF	RMSE MAE STD
2023 [41]	Ignatius Statue Truck Stair Synthetic Industrial Bottle	Nerfacto Instant-NGP TensoRF	RMSE MAE STD
2023 [42]	Space Object	Instant-NGP D-Nerf	PSNR SSIM LPIPS
2023 [43]	Substation Equipment	COLMAP NeRF Instant-NGP	PSNR SSIM LPIPS speed

**Table 3:** Dataset configuration.

Dataset		Images	Scratch Labels
Our synthetic dataset	1890	Training set: 1512	./.
		Validation set: 378	411
NEU-DET real scratch	300	Training set: 240	./.
		Validation set: 60	121

**Table 4:** Comparison of NeRF-based models.

Models	Training Iteration (ms)	Industrial Object 1						Industrial Object 2		
		Dataset 1: 102 Images Train/Val: 92/10			Dataset 2: 192 Images Train/Val: 173/19			Dataset 3: 192 Images Train/Val: 173/19		
		PSNR	SSIM	LPIPS	PSNR	SSIM	LPIPS	PSNR	SSIM	LPIPS
Instant-NPG	16.192	18.90	0.78	0.57	22.83	0.85	0.46	16.28	0.87	0.59
Nerfacto	24.356	14.90	0.74	0.60	21.48	0.84	0.44	21.58	0.89	0.44
Volinga	23.443	14.04	0.73	0.63	20.65	0.83	0.44	20.38	0.88	0.45
TensoRF	61.196	14.52	0.76	0.64	17.51	0.83	0.5	16.96	0.86	0.58

**Table 5:** Comparison of deep learning object detection models.

Method	Paras (M)	FLOPs (G)	mAP@0.5	mAP@0.5:0.95	Precision	Recall	F1-Score
YOLOv5-n	1.76	4.1	70.4	30.7	73.2	63.7	69
YOLOv5-s	7.01	15.8	85.1	41	84.2	80.3	82
YOLOv6-n	4.63	11.34	87.2	41.1	88.8	83.2	85.9
YOLOv6-s	18.05	45.17	91.3	41.2	92.4	89.1	90.7
YOLOX-n	0.91	1.08	86.97	40.59	-/-	-/-	-/-
YOLOX-s	8.94	26.76	90.6	48.42	-/-	-/-	-/-
YOLOv7-Tiny	6.01	13.2	53.8	20.8	61.4	51.6	56
YOLOv7	37.19	105.1	44.8	13.9	51.1	49.4	50
YOLOv8-n	3.0	8.1	92.7	49.5	92.3	87	89
YOLOv8-s	11.12	28.4	96.4	53	96.3	92	94
YOLOv10-n	2.69	8.2	81.6	40.2	80.2	73.1	76
YOLOv10-s	8.03	24.4	89.4	48.5	86.6	82	84

**Table 6:** Ablation study.

			Train: Real, Val: Real					Train: Real + Synthetic, Val: Real				
Method	Paras (M)	FLOPs (G)	mAP@ 0.5	mA@ 0.5:0.95	*p*	R	F1-Score	mAP@ 0.5	mAP@ 0.5:0.95	*p*	R	F1-Score
YOLOv5n	1.76	4.1	22	7.49	20.2	0.43	25	33.5	11.1	29.3	62.9	34
YOLOv5s	7.01	15.8	54.2	19	52.2	56.9	54	63.4	25	50.8	76	61
YOLOv6n	4.63	11.34	61.7	0.283	61	59.5	60.2	80.5	41.6	70.4	82.6	76
YOLOv6s	18.05	45.17	77.4	41.6	73.6	76	74.8	81.9	45.1	78.2	74.4	76.2
YOLOv8n	3.0	8.1	59.2	28.6	48.8	68.6	56	62.4	28.8	62	62	61
YOLOv8s	11.12	28.4	73.5	33.3	72.3	70.2	70	75	36.2	65	78.5	72
YOLO10n	2.69	8.2	36.7	18	44.7	44.6	41	49.3	21.7	46.4	49.6	49
YOLO10s	8.03	24.4	61.8	29.6	65.9	61.2	61.8	65.3	22.2	63.4	70.1	66

## Data Availability

The NEU-DET dataset can be accessed at IEEE Dataport under the title “NEU-DET” (7 November 2024), https://doi.org/10.21227/j84r-f770.
